# Suppression of chronic inflammation with engineered nanomaterials delivering nuclear factor κB transcription factor decoy oligodeoxynucleotides

**DOI:** 10.1080/10717544.2017.1370511

**Published:** 2017-09-05

**Authors:** Leila Farahmand, Behrad Darvishi, Keivan Majidzadeh-A

**Affiliations:** a Recombinant Proteins Department, Motamed Breast Cancer Research Center, ACECR, Tehran, Iran;; b Genetics Department, Motamed Breast Cancer Research Center, ACECR, Tehran, Iran;; c Tasnim Biotechnology Research Center, Faculty of Medicine, AJA University of Medical Sciences, Tehran, Iran

**Keywords:** NF-κB, inflammation, nanomaterials, decoy oligodeoxynucleotides

## Abstract

As a prototypical pro-inflammatory transcription factor, constitutive activation of NF-κB signaling pathway has been reported in several chronic inflammatory disorders including inflammatory bowel disease, cystic fibrosis, rheumatoid arthritis and cancer. Application of decoy oligodeoxynucleotides (ODNs) against NF-κB, as an effective molecular therapy approach, has brought about several promising outcomes in treatment of chronic inflammatory disorders. However, systematic administration of these genetic constructs is mostly hampered due to their instability, rapid degradation by nucleases and poor cellular uptake. Both chemical modification and application of delivery systems have shown to effectively overcome some of these limitations. Among different administered delivery systems, nanomaterials have gained much attention for delivering NF-κB decoy ODNs owing to their high loading capacity, targeted delivery and ease of synthesis. In this review, we highlight some of the most recently developed nanomaterial-based delivery systems for overcoming limitations associated with clinical application of these genetic constructs.

## Introduction

Overall, inflammation and inflammatory responses stem from activation of multiple signaling pathways in different cell types including tissue resident immune cells and/or recruited ones. These pathways together regulate expression of a wide range of pro- and anti-inflammatory cytokines in response to a wide range of stimuli (Lawrence & Gilroy, 2007). Chronic inflammatory disorders are group of diseases identified by presence of a persistent pro-inflammatory condition mostly together with formation of new connective tissue. Rheumatoid arthritis (RA), inflammatory bowel disease (IBD), cystic fibrosis (CF) and cancer are among the most common disorders associated with chronic inflammation (Cicchitti et al., 2015). The number of patients with chronic inflammatory disorders are rapidly increasing each day and based on an epidemiologic screening, chronic inflammatory disorders are among the leading causes of death worldwide (Yach et al., 2004). As the key regulator of inflammatory responses, nuclear factor kappa B (NF-κB) transcription factor is involved in pathogenesis of various chronic inflammatory disorders. NF-κB is mostly maintained inactive in cytoplasm through binding with an inhibitory protein, IκB (Bradford & Baldwin, 2014). However, in many chronic inflammatory disorders NF-κB is constitutively active (Hoesel & Schmid, 2013), turning it to an attractive target for therapy.

**Figure 1.**
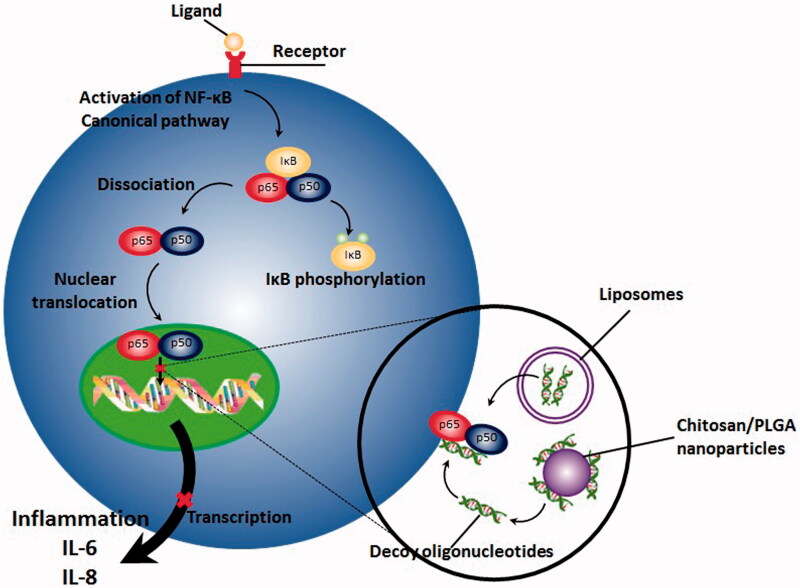
NF-κB decoy ODNs, which have been transferred by different non-viral vectors including nanomaterials result in prevention from translocation of NF-κB, subsequent binding of it with DNA and activation of transcription process.

Gene therapy is referred to the process of transferring nucleic acid into desired cells with the purpose of modifying a medical condition or resulting specific therapeutic outcome (Gascón et al., 2013). Compared to conventional pharmacological inhibitors of NF-κB pathway including non-steroidal anti-inflammatory drugs (NSAIDs) which are mostly nonspecific inhibitors of NF-κB, gene therapy offers number of advantages, especially in the case of diseases with chronic immune-mediated inflammatory nature (Adriaansen et al., 2006). First, it can result in a sustained and prolonged therapeutic outcome without requiring frequent administration of pharmacological agents or therapeutic proteins and second, they can be administered locally at inflammation site, resulting in a significant reduction in side effects and toxicities. As a promising construct in gene therapy, decoy oligodeoxynucleotides (decoy ODNs) are group of double stranded DNA fragments, possessing the same sequence as the binding site of transcription factor on DNA. This occupancy incapacitates subsequent binding of transcription factor to the promoter site of the target genes which in turn, significantly reduces expression of downstream genes (Figure 1) (De Stefano, 2011; Metelev et al., 2013; Johnston & Carroll, 2015).

Application of NF-κB decoys in treatment of several inflammatory disorders have been reported in many studies (De Stefano, 2011; Gasparini & Feldmann, 2012). Investigators have successfully applied NF-κB decoy ODNs for suppressing expression of inflammatory cytokines both *in vitro* and *in vivo* (Griesenbach et al., 2000; Kunugiza et al., 2006; Borgatti et al., 2007; Borgatti et al., 2008; Finotti et al., 2012). Nevertheless, application of these constructs, specially in disorders which require systemic administration, is mostly limited because of two major drawbacks: first, the negative charge of these agents mostly hampers their cellular uptake and second, these short nucleotides are mostly prone to degradation by nucleases and consequently are so unstable (Zaki Ahmad et al., 2013). Regarding these limitations, a clinical trial in which the NF-κB decoy ODNs were administered locally was abandoned due to the lack of efficacy (Fabre & Apparailly, 2011). Consequently, administration of specific vectors for successful delivery of decoy ODNs appears to be inevitable.

Recently, nanotechnology has offered investigators the opportunity for developing several multifunctional carriers for selectively delivering genetical cargos to desired site of action (Kirtane & Panyam, 2013; Nitta & Numata, 2013; Jin et al., 2014). A number of nanocarriers either natural or synthetic including polymers (poly (d,l-lactide co-glicolide) and chitosan), liposomes, gelatin and lipid based nanoparticles have been developed for effective decoy ODNs delivery and some of them have brought about several promising results both *in vitro* and *in vivo*. Furthermore, their ease of production and high-loading capacity has made them valuable candidates for future substitution with currently existing delivery methods.

In the first part of this review we discuss the NF-κB transcription factor’s biology, its pivotal role in pathogenesis of different chronic inflammatory disorders and why decoy ODNs are the best approach for effectively suppressing its activity and then we will review some of the most recently developed nanoformulations for effective delivery of decoy ODNs to the desired site of action for more effective suppress of NF-κB activation.

## NF-κB signaling pathways and its importance in chronic inflammatory disorders

The mammalian NF-κB/Rel family consists of five members including RelA (p65), RelB, c-Rel, NF-κB1 (p50, p105) and NF-κB2 (Panahi et al., 2016). Except for RelB, all members are capable of forming homo- or heterodimers together, each with distinct biological function which has been reviewed elsewhere (Park & Hong, 2016). The most important active form of NF-κB is a heterodimer, consisting of a p50 or p52 subunit and the transactivation subunit p65 (Xia et al., 2014). In its inactive form, NF-κB is presented in cytoplasm in association with eight regulatory proteins referred as IκB. Since IκB proteins have specific tissue distribution pattern and specificities for NF-κB proteins, they can be considered as potent targets for specific therapy (Hinz & Scheidereit, 2014). In most cases, degradation of IκBα results in release and activation of NF-κB. Two main signaling pathways result in activation of the NF-κB, i.e. the canonical and non-canonical pathways (Ghosh et al., 2012).

**Figure 2.**
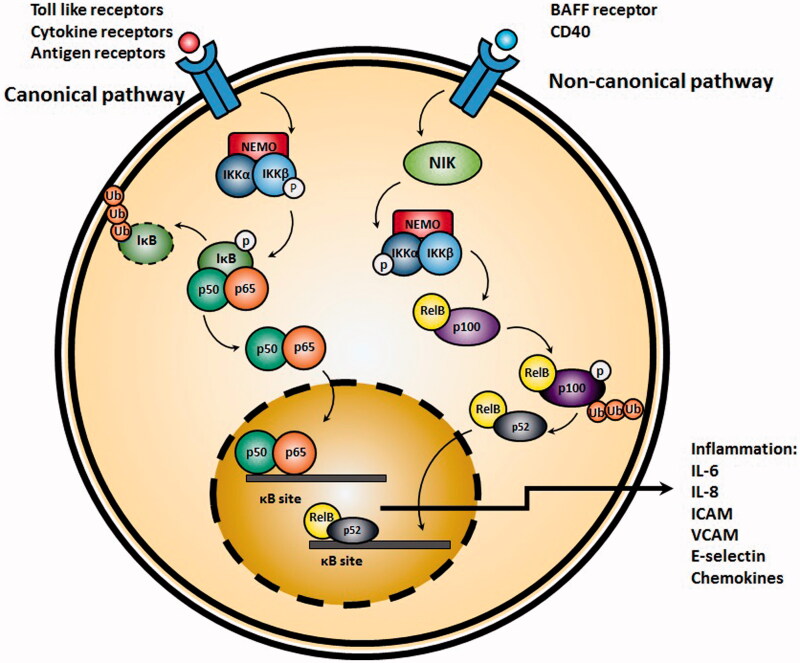
Activation of canonical NF-κB pathway. TNFα or other pro-inflammatory cytokines result in activation of IKK, which in turn, phosphorylates and inactivates Iκβ. Afterwards, NF-κB translocates to the nucleus and initiates transcription of target genes resulting in production of pro-inflammatory cytokines such as IL-6 and IL-8. On the right side, the non-canonical pathway begins by activation of NF-κB inducing kinase (NIK) and formation of p52-RelB dimers which in next step translocate into nucleus.

Initiation of the canonical NF-κB pathway (also referred as classical NF-κB pathway) requires activation of inhibitor of Kβ kinase (IKK) complex, consisting from two catalytic IKKα and IKKβ subunits and a regulatory IKKγ (also referred as NEMO) subunit. Following activation of IKK complex, IκB becomes degraded and results in translocation of the NF-κB dimer p50-RelA to the nucleus. Main inducers of canonical NF-κB pathway include Toll-like receptors, antigen receptor and TNFα receptor signaling pathways. The main activator of the canonical NF-κB pathway in response to the related stimuli is the IKKβ (Tas et al., 2009; Ghosh et al., 2012; Hinz & Scheidereit, 2014). Contrarily, in non-canonical pathway, IKKα homodimers are mostly responsible for activation of NF-κB. IKKα homodimers mostly target NF-κB2/p100 and cause incomplete degradation of this complex to p52 through activation of NF-κB inducing kinase (NIK) and formation of p52-RelB dimers which in next step translocate in to nucleus. Main triggers of this pathway are B-cell activating factor belonging to TNF family (BAFF) receptors and CD40L (Sun, 2011) (Figure 2).

Studies have demonstrated that activation of NF-κB enhances gene expression of different cytokines, chemokines, adhesion molecules and enzymes involved in chronic inflammatory disorders. For instance, increased expression of inducible nitric oxide synthase which is involved in pathogenesis of asthma (Prado et al., 2011), ulcerative colitis (Dhillon et al., 2014) and inflammatory joints (McCartney-Francis, 1993) is a consequence of activation of NF-κB pathway in related cells including macrophages, colonic epithelial cells and synovial cells, respectively (Hatano et al., 2001). Cyclooxygenase II overexpression is another example of NF-κB transcription factor activation. Activation of this inducible enzyme is together with increased expression of prostaglandins and thromboxanes, both of which play pivotal role in pathogenesis of different inflammatory disorders (Tilley et al., 2001; Ricciotti & FitzGerald, 2011). NF-κB also increases activation of several adhesion molecules including intracellular adhesion molecules (ICAM), vascular cell adhesion molecule (VCAM) and E-selectin, all of which effectively involve in recruitment of different inflammatory cells including eosinophils, neutrophils, and T lymphocytes to the inflamed tissues (Bullard, 2002). Increased expression of different pro-inflammatory cytokines including IL-1β, TNF-α, IL-6, granulocyte macrophage colony stimulating factors and many other chemokines taking part in different inflammatory disorders including asthma, inflammatory bowel disease (IBD), psoriasis and rheumatoid arthritis (RA) has also been attributed to the activation of NF-κB (Aggarwal et al., 2004). Additionally, blockade of TNF-α, an important stimulatory factor for activation of NF-κB pathway with antibodies, have shown to control refractoriness of disorder (Elliott et al., 1994). Oxidative stress, another important factor in exacerbating inflammation is also another powerful inducer of NF-κB activation (Morgan & Liu, 2011).

## Decoy oligonucleotide therapy promises and challenges ahead

As a group of recently developed therapeutic agents, transcription factor decoy ODNs have demonstrated remarkable potential in overcoming multiple groups of disorders. These opportunities partly come from capability of these constructs in modulating expression of different genes involved in pathogenesis of these disorders (Mann, 2005). Although ‘gene therapy’ has hardly met some of its speculated promising effects in treatment of different disorders, administration of transcription factor decoys is together with much more advantages compared to gene transfer strategies and also the conventional pharmacological approaches. For instance, transcription factors decoy ODN’s delivery does not require technical challenges associated with obtaining and modifying transfected genetic construct’s expression and is contrarily homologous to the developed straightforward pharmacotherapies. Additionally, for establishment of a successful decoy therapy, only determination of the consensus sequences mostly diagnosed by transcription factors within the gene’s promoter region is enough. Once these sequences are in hand, one can design and examine the effectiveness of these decoys in a couple of weeks, rather than perhaps years required for the development of small therapeutic molecules or the very complex, unpredictable and time-consuming process of determining and developing effective anti-sense agents (Mann, 2005). Currently, the most broadly applied constructs for gene therapy are small interfering RNAs (siRNA) and short hairpin RNAs (shRNA) resulting in temporal downregulation of transcription factors activity (Deng et al., 2014). As these methods mostly restrict production of new transcription factors, existing ones may continue activity until becoming digested by different pathways, resulting in a gradual reduction in transcription factors activity. Contrarily, as decoy ODN are capable of reducing activity of existing transcription factors these constructs result in initiation of effect in a sharper manner (Funabashi et al., 2013).

Unfortunately, just like other therapies, transcription factor decoy ODNs possess their own limitations too. This becomes more important when the transcription factor is involved in regulation of a wide range of cellular restrictions resulting in development of severe toxicities. Nevertheless, this drawback is also associated with administration of nonselective small therapeutic molecules in treatment of chronic inflammatory disorders too. The most handful approach for overcoming this limitation, however, is to selectively target specific transcription factors which are relatively specific in regulating cellular activities including NF-κB in the case of inflammation or E2F in the case of proliferative disorders (De Stefano et al., 2010). Furthermore, application of tissue specific delivery systems, recently offered by rapidly growing field of nanotechnology, is another solution for this drawback. On the other hand, similar to other macromolecules, specifically ODNs, limited cellular uptake is another restricting point for clinical administration of these agents. This mostly results from both ODNs negative charge and large size (Morishita, 2004). Besides, effective *in vivo* delivery of these macromolecules is another important challenge regarding their clinical administration. Although smaller in size compared to nude plasmid DNA, these genetical constructs cannot take advantage of benefits granted by viral delivery systems, both for cell internalization and further translocation to the nucleus where they take action. Additionally, as in most cases ODNs are uptaken by endocytosis, a large part of them will undergo lysosomal degradation (Zaki Ahmad et al., 2013). Fortunately today, different strategies have been developed for improving internalization of ODNs and directing them to cytoplasm instead of endosome-lysosome pathway among which cationic liposomes, lipid-viral particle complexes, covalent linkers and non-covalent carriers have shown lots of promises. Furthermore, development of novel nano-delivery systems has brought about several advantages in overcoming these challenges which has been discussed comprehensively in following sections.

## Why decoy oligonucleotides for suppressing NF-κB pathway?

So far, several studies have reported successful administration of NF-κB decoy ODNs in treatment of a number of inflammatory disorders. The concept of administering a double stranded decoy ODN for suppressing activity of NF-κB was first proposed by Bielinska and colleagues demonstrating that ODNs possessing a κB consensus sequence could effectively compete with DNA in coupling with the NF-κB transcription factor, further inhibiting gene expression (Bielinska et al., 1990). The mechanism underlying this inhibitory effect comes from the fact that decoy ODNs mimic complementary sequence of DNA, through which can bind with NF-κB and prevent it from coupling with cis-element of the specific target gene and initiation of transcription process. After this early report, many investigators begin to examine the therapeutic potential of these genetical constructs for suppressing several disorders associated with chronic inflammation. The potential of NF-κB decoy ODNs in regulating immune responses was first reported by Morishita and coworkers (Morishita et al., 1997). They hypothesized that as NF-κB is the key factor in regulating expression of different genes involved in the production of pro-inflammatory cytokines including IL-6 and IL-8 and expression of adhesion molecules such as ICAM, VCAM and epithelial leukocyte adhesion molecule (ELAM) all of which are involved in the formation of ischemia-reperfusion myocardial injury, suppressing its activity may result in prevention from myocardial infarction. Novel work of Morishita and colleagues demonstrated that NF-κB decoy ODNs transfection to the arteries by hemagglutinating virus of Japan (HJV) liposomes could prevented from induction of myocardial infarction after reperfusion. Alongside, *In vitro* studies demonstrated a significant decrease in expression of VCAM and IL-6 (Morishita et al., 1997). In another study, similar NF-κB decoy ODNs loaded HJV liposomes were applied in a collagen-induced rheumatoid arthritis model for investigating possible immunosuppressive effects. Interestingly, intra-articular administration of these ODNs not only resulted in suppression of IL-1 and TNF-α expression in arthritic joint synovium, but also significantly prevented arthritic joint’s destruction (Tomita et al., 1999). Administration of NF-κB decoys in cystic fibrosis also resulted in significant suppression of IL-8 expression, a key pro-inflammatory cytokine taking role in development of chronic airway inflammation (Griesenbach et al., 2000). Nakamura et al. successfully administered NF-κB decoy ODNs topically for treatment of a murine atopic dermatitis. They demonstrated that NF-κB decoy ODNs resulted in suppression of eczema and induction of apoptosis in dermis infiltrated mast cells (Nakamura et al., 2002). Other study performed by Fichtner-Feigl et al. demonstrated potent anti-inflammatory effects of NF-κB decoys in murine IBD model through reducing expression levels of different pro-inflammatory cytokines including IL-12, IL-23, IL-4, TGF-β and IFNγ from the lamina propria cells and induction of apoptosis in CD4^+^ T cells. Additionally, expression of monocytes derived chemokine was reduced while secretion of IL-10, an inhibitory cytokine was increased (Fichtner-Feigl et al., 2005). Along with these successful outcomes, several other outstanding aspects of NF-κB decoy ODNs therapy has also attracted researcher’s attention for further application of this method in treatment of other chronic inflammatory disorders: first, the target (i.e. the NF-κB transcription factor) is abundant and is also easily detectible in cells. Second, design of sequence-specific decoys are relatively easy and their targeting into particular tissues are easy. Third, identifying the exact sequence of NF-κB transcription factor is not necessary and fourth, in the case of factors being constitutively expressed or when multiple transcription factors negotiate with one similar cis-element, decoy ODNs are more effective in blocking expression of the gene (Morishita, 2004).

## Why nanomaterial based delivery for decoy oligonucleotides?

Overall, as discussed earlier, administration of decoy ODNs in treatment of disorders is mostly hampered due to the extremely poor pharmacokinetic, mostly due to their large size and polyanionic nature, necessitating them to be administered through invasive routes. Furthermore, degrading function of nucleases in biological fluids, rapidly inactivates decoy ODNs and render a very short biological (circulating) half-life to them (Dass, 2002; Fattal & Barratt, 2009; Wang et al., 2015). Decoy ODNs are commonly transferred to the cells by clathrin-mediated endocytosis, most likely through coupling with receptors which interact with anionic molecules such as heparin. Nevertheless, most of them degrade upon introduction to endolysosomes and merely a minute portion of these biomolecules finally gain access to reach cytoplasm and subsequently to the nucleus. Accordingly, the main challenge facing therapies based on decoy ODNs is to prevail over the poor pharmacokinetic profile. For this reason, numerous types of delivery systems have been established to effectively defend decoy ODNs against the destructive function of nucleases and to enhance their permeation in to the cell and cytoplasm with least possible degradation amount (Wang et al., 2015). Several unique properties of nanocarriers including biocompatibility, biodegradability, physical stability in blood and other body fluids, lack of immunogenicity and induction of sustained and prolonged gene expression have made them to be considered as ideal gene vectors (Wong et al., 2017).

Nanotechnology-based systems for decoy ODN delivery generally consists of biocompatible systems including nanoparticles and nanospheres, which can be made from both natural and synthetic materials, cationic solid lipid nanoparticles and nano-sized cationic liposomes which have been successfully applied for improving the pharmacokinetic profile of decoys (Panyam & Labhasetwar, 2003; Morachis et al., 2012). All mentioned delivery systems have shown to effectively enhance internalization of decoy ODNs and protect them from further nuclease enzymatic degradation. Additionally, some of the unique characteristics of NPs have made them privileged for decoy ODN delivering. First, they possess almost similar size as proteins do. Second, they possess large surface area, allowing them to be easily functionalized by different surface functional groups and third, particle size and surface properties of these particles can be easily modified (Nitta & Numata, 2013). More importantly, NPs can be coated with multiple types of molecules and from hydrophilic layers (for instance, PEGylation) further improving their circulating half-life in blood. Modification with other polymers including poloxamers, chitosan and poloxamines can block the hydrophobic and electrostatic interactions of NPs with serum proteins and their opsonization. Application of these polymers can also selectively delivery NPs to their site of action, enhance cellular uptake and internalization of NPs through the receptor-mediated endocytosis and further bypass endolysosomes (Danhier et al., 2012). Accordingly, this flexibility in design and characteristics of NPs have made them to be considered as one of the main candidates for decoy ODN delivery in the treatment of different inflammatory disorders.

## The mechanism of nanocarriers bearing ODN uptake and trafficking in cells

**Figure 3.**
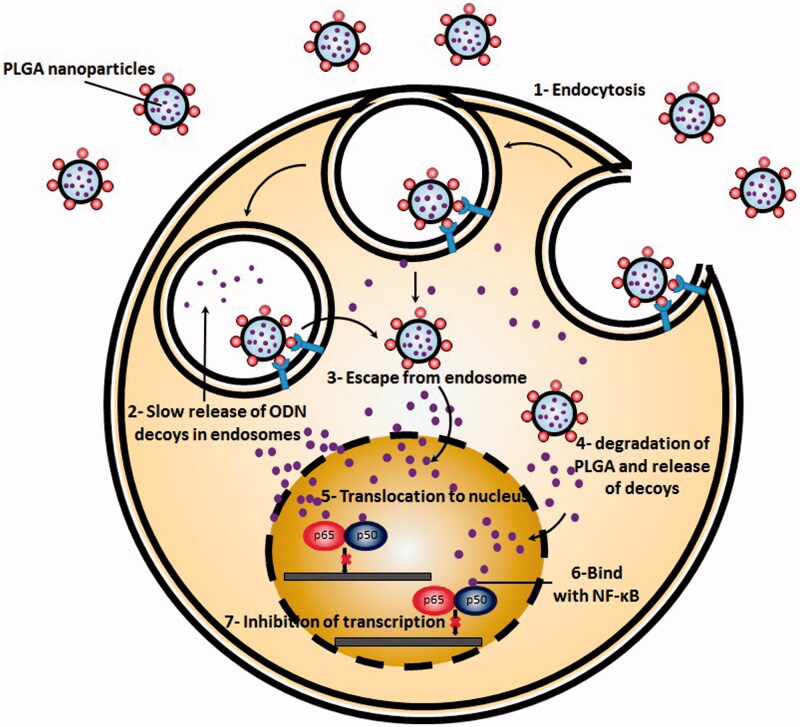
Simplified scheme of NF-κB decoy ODNs delivery by engineered PLGA nanoparticles to the cells. PLGA nanoparticles are first, internalized through endocytosis. In next stem, they escape from endosomes and rich cytoplasm. There they degrade and release decoy ODNs. After translocation to the nucleus, NF-κB decoy ODNs bind with NF-kB and halt transcription process.

Presented at the cell surface, nanocarriers containing ODNs commonly pass through the similar pathway as ‘free’ or conjugated ODNs go (Figure 3). In the first step, they are internalized through endocytotic pathways and then, will be trafficked by different membrane bound intracellular compartments (Juliano, 2016). A number of these membrane bound intracellular compartments include early endosomes, late endosomes, recycling endosomes, Golgi apparatus and endoplasmic reticulum. Accordingly, most of the internalized ODNs either in the free form or in association with nanocarriers are isolated from nucleus and cytosol by cell membrane. In the next step, the endocytotic pathways lead in development of early/re-cycling endosome compartment. Most of the internalized nanocarriers containing ODNs are then accumulated in the endosomes/multivesicular bodies and lysosomes (Juliano, 2016). Additionally, a small portion of nanocarriers are trafficked to the other membrane bound compartments. After endocytosis, accumulated nanocarriers bearing ODNs undergo multi-faceted trafficking pathways each of which can lead to a distinct intracellular destination (Juliano et al., 2013). This complex trafficking network functions in a way to precisely deliver both endogenous and exogenous materials to the most proper position. For instance, a group of bacterial toxins have shown to undergo retrograde transport and traffic to the trans-Golgi compartment where they shift to the cytoplasm (Pfeffer, 2011). The ODN delivery systems also follow a similar goal, to release ODNs from membrane bound compartments and further reach to the cytosol and nucleus.

Rab protein family is the key factor involved in controlling and regulating different aspects of intracellular trafficking. This protein family consists of over 60 members which can both spatially and temporally modulate membrane trafficking and signaling (Schwartz et al., 2007; Stenmark, 2009). Activated forms of Rab GTPases take role as molecular scaffolds which modulate different steps of membrane trafficking including vesicle budding, cytoskeletal transport and targeted docking and fusion. Afterward, nanocarriers bearing ODNs must become released from endosomal vesicles and enter to the cytoplasm. Administration of endosomal disturbing agents have shown to significantly enhance the delivery through this step without affecting overall cellular uptake (Kwon et al., 2008; Detzer et al., 2009). Although nuclear entrance is not a rate-limiting step for free ODNs presented in cytoplasm (Fisher et al., 1993), in the case of ODNs which are mediated in nanocarriers, this step represents a rate limiting step due to the large size of administered nanocarriers. As a result, in order to reach to their site of action, first ODNs must be released from their nanocarrier and once presented in cytoplasm translocate to the nucleus.

## Currently existing delivery systems for NF-κB decoy ODNs

So far, cationic liposomes are the most frequently applied delivery systems for decoy ODNs, characterized by a lipid bilayer encompassing one or more cavities. Cationic liposomes, mostly composed of different cationic lipids, form different complexes with negatively charged ODNs referred as Lipoplexes. Applying optimized conditions during synthesis, different nano-sized and mono-dispersed Lipoplexes are achievable (Jääskeläinen et al., 1994; Jääskeläinen et al., 1998; Jääskeläinen et al., 2000; Derosa et al., 2008). These Lipoplexes are mainly uptaken through clathrin-mediated endocytosis, further becoming destabilized upon fusion with endosomal membrane and diffusing their cargos in to the cytoplasm (Zelphati & Szoka, 1996a,b). *In vitro* Lipoplex-based studies are usually carried out in serum free media to avoid aggregation (Zelphati et al., 1998) and *in vivo* studies with Lipoplexes have been mainly focused on local delivery of NF-κB decoy ODNs. In the study performed by Ono et al. administration of decoy ODN containing Lipoplexes to the CSF of rabbits with cerebral angiopathy following subarachnoid hemorrhage was together with inhibition of cerebral vasospasm and restriction of vessel walls morphological changes (Ono et al., 1998). In another study performed on an experimental renal transplantation model, exposing renal allographs before transplantation for 30 min with NF-κB decoy ODNs complexed with cationic liposomes, resulted in significant decrease in expression of pro-inflammatory genes and significantly suppressed inflammatory responses (Vos et al., 2000). Systemic administration of NF-κB ODN decoys complexed with cationic liposomes has shown to be together with rapid localization in lungs, followed by gradual reposition in liver. In these LPS-treated mice, NF-κB ODN decoys significantly reduced TNFα serum concentrations (Higuchi et al., 2006). In recent studies, cationic liposomes have been applied for specific delivery of NF-κB decoys to splenic macrophages and Kupffer cells utilizing sugar-containing cationic liposomes. The main purpose of these targeted therapies was to overcome immune responses together with suppressing adeno-virus-induced hepatotoxicity (Huang et al., 2009).

Results of the study performed by Higuchi et al. clearly demonstrated that targeted delivery of NF-κB decoy ODNs by fucosylated cationic liposomes (Fuc-liposomes) to the Kupffer cells was together with significant inhibition of pro-inflammatory cytokines production by these cells. They demonstrated that upon IV administration, Fuc-liposome complexes bearing NF-κB decoys were instantly accumulated in liver and internalized by non-parenchymal cells. Additionally, concentrations of IFNγ, TNFα, alanine aminotransferase (ALT) and aspartate aminotransferase (AST) in serum of LPS-infected mice treated with Fuc-liposome complexes were significantly lower compared to the ones treated with unmodified NF-κB ODN decoys (Higuchi et al., 2006). In another study, Wijagkanalan et al. revealed that mannosylated-cationic liposomes loaded with NF-κB decoys were significantly and in high amounts deposited in lungs after intratracheal administration, demonstrating significant inflammation suppressing effects (Wijagkanalan et al., 2011).

In a recent study, a group of nano-vectors formed from mixing protamine, cationic liposome and DNA under optimized condition, were successfully applied for delivering NF-κB decoy ODNs to the lungs upon systemic administration (Tan et al., 2002). Other strategy for further improving delivery of NF-κB decoy ODNs include engraftment of hemagglutinating virus of Japan (HJV) fusigenic proteins to the membranes of liposomes (Kaneda, 2003). The main advantage of these HJV liposomes is bypassing the endocytosis process and direct delivery of decoy ODNs to the cytoplasm. Administration of these liposomes loaded with NF-κB decoy ODNs in the case of a mouse nephritis model, resulted in significant suppression of inflammatory process due to efficient delivery of decoys (Tomita et al., 2000). Administration of HJV liposomes in synovial tissues/cells of rheumatoid arthritis (RA) patients also demonstrated similar results (Tomita et al., 2000). The promising anti-inflammatory effects of these cationic liposomes bearing NF-κB decoy ODNs has also been demonstrated in prevention from liver injury and fibrosis (Son et al., 2007). Along these *in vivo* promising advantages, however, further studies investigating the potential of these agents in inducing immunogenicity seems to be critical as these proteins are exogenous in nature.

Another class of delivery systems for decoy ODNs include polymer-based systems among which polyethyleneimine (PEI), a cationic polymer available in different molecular weights and linear or branched forms, has attracted lots of attentions (von Harpe et al., 2000). Upon electrostatic interaction between decoy ODNs and PEI, polyplexes are formed which can easily internalize in cells through endocytosis (Boussif et al., 1995). PEI possess a high ‘transfection efficacy’ which is mostly ascribed to its ‘proton sponge effect’ also known as ‘buffering effect’ (Godbey et al., 1999). Based on Fischer and colleagues, administration of ODNs complexed with PEIs was together with longer circulation half-life and lower clearance rate compared to free decoy ODNs (Fischer et al., 2004). Modification of PEI with poly ethylene glycol (PEG) has shown to significantly improve circulation half-life and reduce the size of polyplexes containing NF-κB decoys without affecting this polymers’ buffering effect (Kunath et al., 2002; Glodde et al., 2006). Following IV administration of polyplexes, regardless of the type of PEI, liver was the main distribution site of these particles, followed by a negligible amount in kidneys and spleen (Kunath et al., 2002).

Another approach for reducing administration frequencies and enhancing stability of ODNs is to encapsulate them in bio-degradable polymeric platforms. Due to their high biocompatibility, bioavailability, biodegradability and safety profile, poly lactic acid (PLA) and poly (lactic-co-glycolic acid) (PLGA) polymers have been widely investigated as decoy ODN delivery platforms (Athanasiou et al., 1996). The nano- and micro-formulation of these polymers also allow them to be easily injected in to the target tissue without further need for surgical interventions. Different studies have utilized PLA and PLGA for encapsulation of NF-κB decoy ODN release and subsequent development of a prolonged inhibition of NF-κB transcription factor for more than one month (De Rosa et al., 2005). It has been shown that the inhibitory activity of NF-κB decoy ODNs is not affected by the encapsulation process (Gill et al., 2002; De Rosa et al., 2005). Subcutaneous administration of these microspheres loaded with NF-κB decoy ODNs suppressed inflammation up to 15 days in a rat model of chronic inflammation. However, bare decoys with same administration doses could only restrict inflammation for 1 to 5 days (De Stefano et al., 2009).

Gold nanoparticles and dendrimers have also been used for effective delivery of NF-κB decoy ODNs. Rosi et al. Demonstrated that ODNs which were modified by gold nanoparticles possessed higher affinity to the target, higher stability in the presence of nucleases and higher internalization rate compared to unmodified ODNs (Rosi et al., 2006). In the study performed by Sugao et al. a 6th generation dendritic poly (l-lysine) KG6 was applied to deliver NF-κB decoy ODNs to the liver as a treatment for hepatitis. Application of KG6 resulted in significant reduction in serum concentrations of AST, ALT and different inflammatory process related proteins and cytokines (Sugao et al., 2009).

Emergence of nanotechnology platform has resulted in development of several multifunctional delivery materials for selectively delivering genetical cargos to desired site of action (Kirtane & Panyam, 2013; Nitta & Numata, 2013; Jin et al., 2014). A number of nanocarriers either natural or synthetic including polymers (poly (d,l-lactide co-glicolide) and chitosan), liposomes, gelatin, and lipid-based nanoparticles have been recently developed for effective decoy ODNs delivery and some of them have brought about several promising results both *in vivo* and *in vitro*. Furthermore, the ease of production and high-loading capacity has made them valuable candidates for future substitution with currently existing delivery methods.

## Novel engineered nanomaterial-based NF-κB decoy ODN delivery systems

### Chitosan and PLGA-based nanoformulations for NF-κB decoy ODN delivery

PLGA microspheres have shown to significantly increase stability of ODNs in biological media and prolong release profile of these constructs (De Stefano et al., 2009). From other side, it has been clearly demonstrated that chitosan or chitosan modified nanoparticles can be rapidly uptaken by cell culture. Consequently, these two polymers or their combination have been vastly applied for delivering different ODN decoys to transcription factors specially NF-κB (Tahara et al., 2009).

Ulcerative colitis (UC) is considered as a chronic disorder, causing inflammation, severe irritation and sores in the inner facing of the large intestine, in which the pathologic role of NF-κB signaling pathway activation have been clearly identified. In a study performed by Tahara et al., a poly (d,l-lactide co-glicolide) (PLGA) nanospheres were coated with chitosan for enhancing delivery of decoy ODNs both *in vivo* and *in vitro* for suppressing inflammation in ulcerative colitis. Decoy ODNs were then loaded in modified PLGA nanospheres applying an emulsion solvent diffusion method. Chitosan modified PLGA nanospheres demonstrated a positive zeta potential whereas naked PLGA nanospheres where negatively charged. The uptake of nanospheres were further evaluated in Caco-2 cell lines utilizing confocal laser scattering microscopy. Interestingly, chitosan-PLGA nanospheres were more effectively uptaken compared to intact PLGA nanospheres and the stability of decoy ODNs were significantly improved in acidic medium, like gastric juice or at the presence of DNase I enzyme. *In vivo* studies in a rat UC model, demonstrated that daily administration of chitosan-PLGA nanospheres loaded with NF-κB decoy ODNs significantly improved dextran sulfate sodium induced diarrhea. Bloody feces, colon length shortening and the activity of myeloperoxidases. Additionally, decoy ODN-loaded chitosan-PLGA nanospheres were shown to more effectively deposited and adsorb on the inflamed ulcerative colitis model rats mucosal tissue. Based on results, authors concluded that oral administration of NF-κB decoy ODN-loaded chitosan modified PLGA nanospheres represent a promising carrier for treatment of ulcerative colitis (Tahara et al., 2011).

As NF-κB plays pivotal role in the pathogenesis of cystic fibrosis, silencing this pathway may be a potential target for therapy. In a study performed by Wardwell et al., polysialic acid-N-trimethyl chitosan polymers (PSA-TMC) were applied for preparation of nanoparticles which were then coated with NF-κB decoys. To evaluate the anti-inflammatory effect of NF-κB decoy ODNs coated nanoparticles, an in vitro model which was generated via treatment of IB3-1 bronchial epithelial cells with lipopolysaccharides of pseudomonas aeruginosa or Interleukin-1β (IL-1β). Whilst treatment of epithelial cells with intact decoy ODNs and scrambled ODN coated nanoparticles did not result in any significant changes in secretion of pro-inflammatory mediators of cystic fibrosis, NF-κB decoy ODN-coated PSA-TMC nanoparticles significantly decreased secretion of IL-6 and IL-8 from epithelial cells, particularly when the treatment period was increased. However, the *in vivo* safety and efficacy of these nanoparticles were not analyzed in this study (Wardwell & Bader, 2015).

NF-κB has also been shown to be involved in pathology of rheumatoid arthritis (RA), an autoimmune disorder which is identified by imbalancement between pro- and anti-inflammatory cytokines. Similar group also applied PSA-TMS-ODN in RA to improve stability of ODN decoys and cellular uptake of them. The study performed on two *in vitro* models, a primary RA cell model and a cell line based model. Like previous study on cystic fibrosis, bare ODN decoys could not significantly decrease secretion of pro-inflammatory cytokines including IL-6 and IL-8. However, PSA-TMS-ODNs sufficiently suppressed secretion of pro-inflammatory cytokines. Also utilizing fluorescence microscopy, sufficient cellular uptake of PSA-TMC nanoparticles were confirmed (Wardwell et al., 2015).

In another study, de Stefano et al. prepared large porous particles of PLGA in an inhalable dry powder form for evaluating the inhalable NF-κB decoy ODNs efficacy in treatment of cystic fibrosis *in vivo*. Loaded large porous PLGA particles with decoy ODNs were engineered to achieve proper aerodynamic characteristics, acceptable loading, prolonged release and preserved integrity in biological fluids lining the pulmonary tract. The *in vivo* cystic fibrosis models were rats with lung inflammation induced by intratracheal aerosolization of *Pseudomonas aeruginosa* LPS. Based on results, a single inhalation of large porous decoy ODNs-loaded PLGA particles could significantly reduce PLS induced bronchoalveolar infiltration of neutrophils up to 72 h. while bare decoy ODN could only inhibit it for 6 h. Additionally, they also demonstrated that persistent neutrophil infiltration inhibition resulted in reduced production of IL-6 and IL-8 and suppression of mucin 2 mRNA expression in lung homogenates. Consequently, the authors concluded that lung porous PLGA nanoparticles were proper carriers for local treatment of inflammation in lungs by NF-κB decoy ODNs (De Stefano et al., 2013).

Pulmonary arterial hypertension (PAH) is considered as an intractable, highly lethal disorder of the small pulmonary artery with significant inflammatory nature and it has been assumed that NF-κB is the pivotal transcription factor, regulating secretion of different inflammatory cytokines involved in pathogenesis of this disease. Based on immunohistochemical results, activity of NF-κB in small pulmonary arterial lesions and macrophages of patients suffering from PAH is significantly higher compare to normal patients, making NF-κB a proper target for proper treatment of PAH. Kimura et al. developed a poly ethylene glycol (PEG)-PLGA nanoparticle and encapsulated decoy ODNs in them utilizing an emulsion solvent diffusion technique with average particle size of 44 nm. Single intratracheal instillation of these nanoparticles in monocratiline- induced PAH rat models resulted in presence of nanoparticles for up to 14 days after instillation in lungs of rats. Presence of nanoparticles loaded with NF-κB decoy ODNs in lungs significantly suppressed pulmonary inflammation induced by monocratiline and significantly attenuated development of PAH. Additionally, administration of NF-κB decoy ODN loaded nanoparticles 3 weeks after injection of monocratiline could also significantly improve the survival of NF-κB pathway in development of PAH and prolonged suppression of this pathway by administration of nanoparticle as a proper strategy against PAH (Kimura et al., 2009).

### Liposomal based delivery systems for NF-κB ODN decoys delivery

Although still a much higher efficacy and lower toxicity is required for liposomes to be applied as effective delivery system for decoy ODNs, they are currently widely applied to improve cellular uptake of ODNs in different cells. In a study performed by de Rosa et al. a new liposomal formulation based on a recently synthetized cationic lipid (2,3-didodecyloxypropyl)(2-hydroxyethyl) demethylammunium bromide (DE) was prepared for evaluating its potential for delivering double-stranded decoys against NF-κB to LPS-stimulated RAW 264.7 macrophages and the potency of them in suppressing NF-κB activation. In the formulation of liposomes, 1, 2-dioleyl-sn-glycero-3-phosphoethanolamine or cholesterol were applied as helper lipids. Either liposomes alone or mixed with helpers were complexed with ODNs at different ± charge ratios. Among formulations, DE/cholesterol mixed liposomes which were complexed with ODNs at ± charge ratios equal to 8, demonstrated highest inhibition rate of nitric production, highest suppression of inducible nitric oxide synthase protein expression and lowest binding of NF-κB to DNA. Also, confocal microscopy demonstrated a high cellular uptake of ODNs when DE/cholesterol liposomes where applied at the highest ± charge ratios (Derosa et al., 2008).

In the study performed by Buchanan et al., a new type of liposome, the ‘echogenic liposome’ which co-encapsulates a gas together with drugs capable of releasing cargo upon application of ultrasounds, was applied for delivering NF-κB ODN decoys. Application of this technique brought about three major advantages. First, packing in liposomes may significantly protect ODNs from degradation. Second, drug cargos can be easily and in a desired manner release upon application of ultrasound. Through this, high amplitude ultrasound pulses result in a bolus release of ODNs, low amplitudes may be applied for sustained release and finally, combination may be applied for therapeutic usage. Third, the ultra sound driven cavitation of the gas bubbles significantly improves transportation of genes and drugs in to the arterial walls. ODN decoys were incorporated in echogenic liposomes utilizing freeze/thaw technique. Based on results, application of 1 MHz continuous wave, 0.26 MPa peak to peak pressure amplitude for 60 s resulted in release of 41.6 ± 4.3% of ODNs from echogenic liposomes. Since these cargos are easily passed from arterial walls, they are good candidates for application in cardiovascular disorders (Buchanan et al., 2010).

Restenosis after endovascular interventions is considered as one of the main obstacles in cardiovascular disorders treatment which is partly due to the multiple cellular and molecular reactions occurring in the vascular walls after angioplasty. Alongside, absence of an intact endothelial monolayer or endothelial dysfunction, mostly unsettles the homeostatic regulation of thrombosis. Furthermore, accumulation of inflammatory cells together with activation of vascular smooth muscle cells (VSMCs) induced by vascular injury promotes secretion of multiple types of cytokines and growth factors which in turn enable VSCM growth, deposition of extracellular matrix and ultimately development of neointimal hyperplasia (Casscells, 1992; Davies & Hagen, 1994). As NF-κB is mostly involved in regulating inflammation and proliferation and migration of VSCMs (Matsushita et al., 2000; Joyce et al., 2001; De Winther, 2005), silencing of this transcription factor may play an important role in inhibiting development of neointimal hyperplasia. Furthermore, a number of studies have also demonstrated that the NF-κB is activated after angioplasty and also in atherosclerotic vessels (Brand et al., 1996; Bourcier et al., 1997). In the study performed by Miyake et al. an angioplasty balloon coated with chitosan modified PLGA nanospheres, was used to deliver NF-κB decoy ODNs to the vascular walls. These nanospheres were electrostatistically bound to the balloon. They investigated whether application of NF-κB decoy ODN coated balloon catheter could inhibit neointimal formation in rabbit vascular injury model or not. Interestingly, administration of these nanospheres could significantly inhibit NF-κB activity and thereby, neointimal formation. Furthermore, application of nanospheres could also facilitate endothelial cell monolayer restoration through increasing expression of phosphorylated Bcl2 (Miyake et al., 2014). The advantages and drawbacks of each nanodelivery system have been summarized in Table 1.

**Table 1.** Advantages and disadvantages of nanomaterials applied for ODN and gene delivery.

Nanocarrier	Advantages	Disadvantages	References
Echogenic liposomes	Protection against nucleases, controlling release profile through adjusting ultrasound parameters, enhanced gene delivery into the arterial wall	–	(Buchanan et al., 2010)
PEG-PLGA NPs	Sustained ODN release pattern after a single instillation upon degradation of NPs, protection against degradation during intracellular trafficking, decreased opsonin binding, prolonged circulation time	–	(Kimura et al., 2009)
PLGA large porous NPs	Sustained drug release profile, improved localization in particular area of the lungs, facilitated transport across the mucus layer, and improved uptake by epithelial cells	PLGA has a low-release rate and low-encapsulation efficiency in the case of of pDNA; making microenvironment acidic	(Hedley, 2003; De Stefano et al., 2013)
N-trimethyl chitosan-polysialic acid (PSA-TMC) NP	Improving ODN cellular uptake by increased interaction with negatively charged cell membrane, As PSA has no known receptors in the body, it can easily evade RES and decrease immunogenicity. Extension of circulation time	Chitosan in formulation demonstrates low solubility in physiological pH and low-transfection efficiency	(Borchard, 2001; Bozkir & Saka, 2004; Prabaharan & Mano, 2004; Wardwell & Bader, 2015)
Chitosan-modified PLGA NS	Increased mucoadhesion, protection against acidic medium, suitability for oral delivery, prolonged release profile	–	(Tahara et al., 2011)
PEI	Unique DNA condensation capability; unique proton-sponge effect; high buffering capacity and transfection efficiency;	Low biodegradation rate; the inconsistency between transfection efficiency and cytotoxicity;	(Jin et al., 2014; Wang et al., 2016)
SLN	Ease of preparation, safe component materials, low cost, high stability and controlled drug release. Can undergo steam sterilization, lyophilization,	Cytotoxicity due to cationic surfactants, emulsifiers and preservatives in the formulation	(Vighi et al., 2012; Ma et al., 2015)
Cationic liposomes	Biodegradability, biocompatibility, low immunogenicity, simplicity, reproducibility	–	(Serikawa et al., 2008; Dizaj et al., 2014)

## Lipid-based nanoparticles and gelatin nanoparticles for selective delivery of NF-κB ODN decoys to macrophages

Macrophages are the pivotal effector cells in RA due to secretion of several inflammatory cytokines including IL-6 and IL-8. Consequently, selective targeting of NF-κB pathway in these cells is together with several beneficial outcomes. Based on this theory, Hattori et al. prepared folate linked lipid based nanoparticles (NP-F) for delivering NF-κB decoy ODNs in to murine macrophages. As LPS-activated RAW264.7 macrophages strongly express folic acid receptors, modifying nanoparticles with folic acid is an excellent effort for improving selectivity of NPs for these cells. After transfecting NP-F, decoys were clearly detected in cytoplasm of macrophages and 0.03 mM concentration of decoys, resulted a significant inhibitory effect on translocation of NF-κB to the nucleus. Based on results, the authors concluded that the lipid nanoparticles are valuable vectors for delivering decoys to the activated macrophages and subsequent suppression of inflammatory processes in RA (Hattori et al., 2006).

The important point in suppressing NF-κB pathway in liver is that this inhibition may cause both destructive and protective effects simultaneously and the net outcome is mostly depended on the type of cells being suppressed. In the study performed by Hoffmann et al. the effect of NF-κB suppression specially in Kupffer were evaluated applying gelatin nanoparticles. They successfully loaded NF-κB decoy ODNs in gelatin nanoparticles and evaluated their *in vivo* distribution. Afterwards, the liver damage, the levels of pro-inflammatory cytokines, apoptotic proteins expression levels and NF-κB activities were evaluated after LPS, N-galactosamine (GalN)/LPS challenge and partial warm ischemia and subsequent reperfusion. Results demonstrated that decoy loaded nanoparticles were selectively and also sufficiently uptaken by Kupffer cells and NF-κB pathway was subsequently suppressed in these cells. This inhibition significantly increased survival and effectively reduced liver injuries subsequent to the GalN/LPS challenges. Although the expression levels of anti-apoptotic proteins in liver was not significantly affected, pro-apoptotic players including JNK, were significantly reduced. Nevertheless, selective NF-κB suppression in Kupffer cells enhanced the reperfusion injury (Hoffmann et al., 2009).

## Conclusion and future perspective

As briefly discussed in this review, the strategies focusing on inhibiting the activation of NF-κB signal transduction pathway through applying NF-κB decoy ODNs provide a promising tool for more effectively tackling chronic inflammation in retractable chronic inflammatory disorders. However, the ultimate beneficial outcome is mostly resulted from delicate balance between suppressing inflammation and normal cellular function interference. Consequently, application of specific targeted delivery systems may mostly be handful in optimizing interference only in targeted cells. From other side, in order to approach toward clinical application, applying different methods for limiting immune responses to delivery systems, increasing the effectiveness, efficiency of delivering and prolonging the half-life of decoy ODNs in body seems to be crucial. Application of nanomaterials as promising non-viral vectors although in its infancy, has mostly addressed some of the challenges mentioned earlier. Nevertheless, lack of *in vivo* studies for evaluating possible toxicities and selective distribution of these particles in body seems to be an important question which should be answered in future studies.
